# Factors affecting the uptake of prenatal screening tests for congenital anomalies; a multicentre prospective cohort study

**DOI:** 10.1186/1471-2393-14-264

**Published:** 2014-08-09

**Authors:** Janneke T Gitsels - van der Wal, Pieternel S Verhoeven, Judith Manniën, Linda Martin, Hans S Reinders, Evelien Spelten, Eileen K Hutton

**Affiliations:** Department of Midwifery Science, AVAG and the EMGO Institute for Health and Care Research, VU University Medical Centre, Van der Boechorststraat 7 (D4.44), 1081 HV Amsterdam, Netherlands; Faculty of Theology, VU University Amsterdam, Amsterdam, Netherlands; University College Roosevelt, Middelburg, Netherlands; Department Midwifery Education Program, McMaster University, Hamilton, Canada; Department of Medicine, Nursing, and Health Sciences, Monash University, Melbourne, Australia

**Keywords:** Congenital abnormalities, Fetal anomaly scan, Down syndrome, Combined test, Second trimester pregnancy, Religion, Immigrants, Ultrasonography

## Abstract

**Background:**

Two prenatal screening tests for congenital anomalies are offered to all pregnant women in the Netherlands on an opt-in basis: the Combined Test (CT) for Down syndrome at twelve weeks, and the Fetal Anomaly Scan (FAS) at around twenty weeks. The CT is free for women who are 36 or older; the FAS is free for all women. We investigated factors associated with the CT and FAS uptake.

**Method:**

This study is part of the DELIVER study that evaluated primary care midwifery in the Netherlands. Associations between the women’s characteristics and the CT and FAS uptake were measured using multivariate and multilevel logistic regression analyses.

**Results:**

Of 5216 participants, 23% had the CT and 90% had the FAS, with uptake rates ranging from 4% to 48% and 62% to 98% respectively between practices. Age (OR: 2.71), income (OR: 1.38), ethnicity (OR: 1.37), being Protestant (OR: 0.25), multiparous (OR: 0.64) and living in the east of the country (OR: 0.31) were associated with CT uptake; education (OR: 1.26), income (OR: 1.66), being Protestant (OR: 0.37) or Muslim (OR: 0.31) and being multiparous (OR: 0.74) were associated with FAS uptake. Among western women with a non-Dutch background, first generation (OR: 2.91), age (OR: 2.00), income (OR: 1.97), being Protestant (OR: 0.32) and living in the east (OR: 0.44) were associated with CT uptake; being Catholic (OR: 0.27), Protestant (OR: 0.13) were associated with FAS uptake. Among non- western women with a non-Dutch background, age (OR: 1.73), income (OR: 1.97) and lacking proficiency in Dutch (OR: 2.18) were associated with CT uptake; higher education (OR: 1.47), being Muslim (OR: 0.37) and first generation (OR: 0.27) were associated with FAS uptake.

**Conclusion:**

The uptake of the CT and FAS varied widely between practices. Income, parity and being Protestant were associated with uptake of both tests; ethnicity, age and living in the east were associated with CT uptake, and education and being Muslim with FAS uptake. These findings help to explain some differences between women choosing or declining early and late screening, but not the large variation in test uptake among practices, nor between the Netherlands and other countries.

## Background

Since 2007, all pregnant women in the Netherlands are informed about prenatal congenital anomaly screening tests. The available screening consists of two non-invasive tests: the Combined Test (CT) at around twelve weeks to determine the possibility of Down syndrome, and the Fetal Anomaly Scan (FAS) at around twenty weeks’ gestation to detect structural anomalies. Both CT and FAS potentially require three decisions: 1) whether to have (one of) the screening test or not, 2) whether or not to follow up positive screens with diagnostic tests, which carry an associated 0.5% risk of miscarriage and 3) if diagnostic tests confirm a positive finding, whether to terminate the pregnancy before 24 weeks’ gestation or prepare for having a child with an anomaly. In contrast to other countries such as the United Kingdom, Denmark and Iceland, the tests are not routinely offered as part of prenatal care in the Netherlands [1–4]. Although both tests are part of a population based screening programme, they are not offered on the same basis. The CT is free of charge for women who are 36 or older, while younger women choosing to have the test pay approximately 150 euros; the FAS is free for all women.

The mean uptake of prenatal congenital anomaly screening tests in the Netherlands has been around 27% for some years for the CT but varies between different regions (12% to 52%) [5–8]; the mean uptake of the FAS has been around 91% (80% to 99%) [6, 7]. Both tests’ uptake figures are considerably lower than in other European countries [1–4]; in Denmark and Iceland for example, the CT uptake is at least 90% and in Sweden the FAS uptake is up to 100% [3, 4, 9]. Earlier studies of the CT have shown that factors such as age, parity, ethnicity, socioeconomic status, immigrants’ language proficiency and religion can influence uptake decisions [8, 10–19]. However, although both CT and FAS are part of the prenatal congenital anomaly screening programme, little is known about the factors influencing second-trimester FAS uptake. We were also interested in the sub-groups of western and of non-western women with a non-Dutch ethnic background getting care in the Netherlands. In 2010, women with a non-Dutch ethnic background were responsible for 27% of all live births [20] and it is known that immigrant women have made suboptimal use of prenatal care in the past [21, 22]. To understand the differences in uptake and to use this knowledge to ensure that all women have equal access to prenatal anomaly screening, it is important to identify determinants influencing the uptake of the CT and the FAS. Therefore, the present nationwide study investigated factors influencing the uptake of the prenatal anomaly screening tests.

Based on previous studies [8, 10–19] and the fact that both the CT and the FAS are followed by the same diagnostic options for positive results, we hypothesized that CT and FAS screening uptake would be associated with religious background, age, parity, socioeconomic status and ethnicity, and that (except for ethnicity) the same hypotheses plus an association with proficiency in Dutch language would hold for women with a non-Dutch background.

Of all pregnant women in the Netherlands, more than 80% start their prenatal care in primary care midwifery and receive information and counselling about prenatal screening from primary care midwives [23]. This study was therefore undertaken in primary care midwifery practices.

## Methods

### Study design

For this study we used data from the DELIVER study, a multicentre prospective dynamic cohort study evaluating primary care midwifery in the Netherlands. Between September 2009 and February 2011, data were collected from clients and midwives in twenty midwifery practices across the Netherlands. The clients’ response rate at the twenty practices was 58% on average and ranged from 32% to 72%. A complete overview of the design of the DELIVER study was given by Manniën *et al.* [24]. Depending on when clients started prenatal care relative to the timing of the study, clients may have completed one, two or three questionnaires. Questions regarding use of the CT and the FAS were asked in both the second questionnaire (completed between 35 weeks of gestation and birth) and the third questionnaire (completed six weeks post-partum). We used data from all participants who completed either questionnaire; in the case of women who completed both questionnaires, we used their responses to the questionnaire completed between 35 weeks and birth. Socio-demographic characteristics such as parity, consanguinity and Dutch language skills among women with a non-Dutch ethnic background were asked as part of a demographic profile in the first questionnaire completed by a study participant. Privacy was guaranteed in accordance with Dutch legislation. Participants’ anonymity was maintained by using anonymous practice identifiers.

### Measures

In this study, accepting or declining the CT or FAS were the (dichotomous) dependent variables. The demographic data collected included religious background, age, education and after tax income per household. The Netherlands was divided into four regions (north, east, west and south). We used the classification of Statistics Netherlands to determine women’s ethnicity as Dutch or non-Dutch, that is: having at least one parent born in a country other than the Netherlands [25]. For non-western women with a non-Dutch ethnic background, we again used the classification of Statistics Netherlands and included those women from Africa, Latin-America, Asia (except for Indonesian and Japan) or Turkey [26]. A woman was of the first generation non-Dutch if she was born abroad, a woman was of the second generation non-Dutch if (one of) her parents was born abroad. Consanguinity was ascertained by asking the question if her partner is a cousin, uncle or nephew. Participants of non-Dutch ethnic background were asked to rate their Dutch proficiency using a four-point scale (none, a little, fair and excellent) on four items that assessed speaking, understanding, reading and writing Dutch. A woman was nulliparous if she had not given birth before; multiparous pregnant women were those who had given birth before.

### Analysis procedure

In the preparatory analyses, we constructed new variables. Using the Dutch Protestant ecclesiastical map, we constructed a variable in which the different religions and denominations are grouped into the following resulting categories: ‘Catholic’; ‘Protestant’ = Protestant Church, smaller orthodox Calvinist, Evangelical and Pentecostal Churches, Mennonites/Armenians/Episcopalism; ‘Islam’; ‘other’ = Judaism, Buddhism, Hinduism; ‘would not say’ = would not say or do not know; ‘none’ = none, Humanism. Based on the relationship between the age of the mother and the probability of being pregnant with a child with trisomy 21 (Down Syndrome), we divided age into three groups (≤30, 31–35, ≥ 36) [27]. We divided the level of education into three groups (‘low’ = mainly primary school and some vocational training, ‘medium’ = secondary school and completed vocational training, ‘high’ = college and/or university) [28]. We dichotomized income into the following categories: category 1 = none to the mean disposable income; and category 2 = higher than the mean disposable income in the Netherlands in 2010 (1811 euros/month). Disposable income is total income after tax. We constructed a new variable for Dutch proficiency by taking the mean score for speaking, understanding, reading and writing (Cronbach’s alpha 0.98). Additionally, the Dutch proficiency variable was dichotomized into ‘excellent’ and ‘limited’ (none, a little or fair).

Descriptive statistics were used to summarize socio-demographic characteristics. We used univariate logistic regression analyses to obtain odds ratios and 95% confidence intervals to determine if the background characteristics, pregnancy-related variables, Dutch languages skills among women of non-Dutch ethnic background significantly influenced test uptake. We performed *χ*^*2*^ tests in order to examine a possible association between the aforementioned factors and uptakes of the CT and FAS. We used multivariate logistic regression analysis to determine the association between CT and FAS uptake whereby every significant variable (*p* < 0.05) from the univariate tests was added as a predictor. Separate models were analysed for the uptake of the two dependent variables, uptake of the CT and of the FAS (yes/no). Backward selection was performed on the initial multivariate model for the sequential removal of variables: in each step, the variable with the largest *p* value was removed until the model contained only statistically significant variables (two-sided *p* < 0.05).

Next, multilevel analyses were carried out to account for any possible effects due to differences between practices. The likelihood ratio test was used to determine if there was a random intercept for ‘practice’. Random slopes for each variable were then considered to assess whether the influence of the variable was different for different practices. Again, the likelihood ratio test was used to evaluate the benefit to the model of a random slope for a variable. The final logistic regression model for each independent variable was determined using backward selection.

Lastly, we performed a subgroup analysis for non-Dutch women following the same procedures as outlined above excluded multilevel analyses. We used the same variables as for the complete study population, except that we removed ethnicity and added the variables of Dutch language proficiency and generation. STATA 10.0 was used for the multilevel analyses; the other analyses were performed using SPSS 21.0.

## Results

Of the 7,907 participants in the DELIVER study, 5216 women completed questionnaires containing questions about CT and FAS. Comparison with the national perinatal registration revealed that our study population is representative for parity (nulliparous: 47% in our data versus 48% nationwide) and age (aged 34 or younger: 79% versus 78%), but had more highly educated women (51% versus 42%) and fewer ethnic minority women (16% versus 27%) [24].

Table 1 shows background characteristics of the participants in relation to the uptake of the CT and FAS. It also provides information from the univariate logistic regression analyses. The mean actual uptake for the CT was 23% (1,183/5,216), and 90% for the FAS (4,679/5,216). The average CT uptake of women with a non-Dutch background was 29% (237/808), significantly higher in comparison to the native Dutch participants of this study (22%; *p* < 0.001). The average uptake for FAS of women with a non-Dutch background was 89% (720/808), a proportion very similar to the native Dutch participants in the study. The uptakes ranged in the practices from 4% to 48% for the CT and from 62% to 98% for the FAS (Table 2). Table 3 shows the results of the multivariate logistic regression analyses. Cells with small numbers were left out of the analysis.

**Table 1:** **Background characteristics of the study population and univariate logistic regression analyses of the uptake of the combined test (CT) and the fetal anomaly scan (FAS) among pregnant women participating in the DELIVER study**

Determinant	Population	CT Uptake		FAS Uptake	
	N (%) ^a^	N (%) ^b^	OR (95% CI)	N (%) ^b^	OR (95% CI)
**Total**	5,216 (100)	1,195 (23)		4,679 (90)	
**Religion**					
**None**	3,042 (59)	852 (28)	1	2,909 (95)	1
**Islam**	247 (5)	50 (20)	0.66 (0.48-0.90)^c^	198 (80)	0.20 (0.14-0.28)^d^
**Protestantism**	1,210 (23)	74 (6)	0.17 (0.13-0.22)^d^	913 (76)	0.15 (0.12-0.19)^d^
**Catholicism**	547 (10)	166 (30)	1.13 (0.92-1.37)	514 (94)	0.76 (0.52-1.12)
**Other** ^**e**^	37 (1)	17 (46)	2.19 (1.14-4.21)^c^	35 (95)	0.85 (0.20-3.59)
**Would not say**	90 (2)	24 (27)	0.94 (0.58-1.51)	78 (87)	0.32 (0.17-0.60)^d^
**Age (years)**					
**≤ 30**	2,507 (48)	326 (13)	1	2,235 (89)	1
**31-35**	1,872 (36)	463 (25)	2.20 (1.88-2.57)^d^	1,700 (91)	1.20 (0.98-1.47)
**≥ 36**	833 (16)	406 (49)	6.36 (5.32-7.61)^d^	741 (89)	0.98 (0.76-1.26)
**Level of education**					
**Low**	697 (13)	145 (21)	1	575 (82)	1
**Medium**	1,867 (36)	329 (18)	0.81 (0.66-1.01)	1,658 (89)	1.68 (1.32-2.15)^d^
**High**	2,627 (51)	713 (27)	1.42 (1.16-1.74)^c^	2,421 (92)	2.49 (1.96-3.18)^d^
**Ethnic background**					
**Dutch**	4,397 (84)	956 (22)	1	3,948 (90)	1
**Non-Dutch**	808 (16)	237 (29)	1.49 (1.26-1.77)^d^	720 (89)	0.93 (0.73-1.19)
**Income**					
**None to average**	1,752 (41)	291 (17)	1	1,493 (85)	1
**Above average**	2,507 (59)	675 (27)	1.85 (1.59-2.16)^d^	2,338 (93)	2.40 (1.96-2.95)^d^
**Region**					
**West**	1,511 (29)	461 (31)	1	1,360 (90)	1
**East**	1,622 (31)	208 (13)	0.34 (0.28-0.40)^d^	1,371 (85)	0.61 (0.49-0.75)^d^
**South**	739 (14)	226 (31)	1.00 (0.83-1.22)	697 (94)	1.84 (1.19-2.63)^d^
**North**	1,344 (26)	300 (22)	0.65 (0.55-0.78)^d^	1,251 (93)	1.50 (1.14-1.96)d^c^
**Parity**					
**Nulliparous**	2,472 (47)	602 (24)	1	2,298 (93)	1
**Multiparous**	2,739 (53)	592 (22)	0.86 (0.75-0.96)^c^	2,376 (87)	0.50 (0.41-0.60)^d^
**Consanguinity**					
**No**	5,053 (99)	1,154 (23)	1	4,545 (90)	1
**Yes**	58 (1)	11 (19)	0.79 (0.41-1.53)	42 (72)	0.29 (0.16-0.53)^d^

OR = Odds Ratio; CI = confidence interval; ^a^sample size varies due to missing data; valid percentages are shown; ^b^uptake percentage of population per category**;** ^c^p < 0.05; ^d^p < 0.001; ^e^Judaism, Buddhism, Hinduism.

**Table 2:** **Distribution of the average CT and FAS uptake across the midwifery practices**

Practice	CT (%)	FAS (%)	Region
**1**	28	97	South
**2**	37	96	South
**3**	22	87	East
**4**	24	98	West
**5**	48	92	West
**6**	**4**	**63**	**East**
**7**	14	94	North
**8**	22	92	North
**9**	41	94	West
**10**	16	92	East
**11**	13	90	North
**12**	29	96	North
**13**	**5**	**76**	**West**
**14**	11	95	East
**15**	15	94	East
**16**	23	91	North
**17**	**4**	**62**	**East**
**18**	33	89	West
**19**	15	86	South
**20**	42	94	West

Bold rows are practices located in the Dutch ‘Bible Belt’, a predominantly orthodox Protestant region.

**Table 3:** **Multivariate logistic regression analysis of all significant factors affecting test uptake amongst pregnant women participating in the DELIVER study**

	CT Model (n = 4,240)		FAS Model (n = 4,247)	
Predictor	OR (95% CI)	***p***-value	OR (95% CI)	***p***-value
**Higher education**			1.35 (1.16-1.58)	<0.001
**Non-Dutch background**	1.42 (1.13-1.79)	0.003		
**Increasing age**	2.76 (2.47-3.10)	<0.001		
**Protestantism**	0.21 (0.16-0.28)	<0.001	0.21 (0.17-0.27)	<0.001
**Islam**	0.61 (0.40-0.94)	0.023	0.37 (0.25-0.57)	<0.001
**Above average income**	1.43 (1.21-1.70)	<0.001	1.73 (1.37-2.18)	<0.001
**North region**	0.63 (0.51-0.78)	<0.001	1.30 (0.94-1.80)	0.107
**East region**	0.35 (0.28-0.44)	<0.001	0.73 (0.56-0.95)	0.021
**South region**	1.18 (0.93-1.48)	0.171	1.53 (1.01-2.31)	0.030
**Multiparous**	0.64 (0.54-0.76)	<0.001	0.73 (0.61-0.87)	0.001

OR = odds ratio; CT model: −2 log likelihood = 3,771.198, 80%correctly predicted; FAS model: −2 log likelihood = 2,413.095, 90% correctly predicted.

Table 4 shows the results of the multilevel analyses with a random intercept for practice in both the CT model and the FAS model, and additionally, a random slope for the effect of Protestantism in the FAS model. Regarding the CT model (*χ*^*2*^ (9, *N* = 4,240) = 412.63, *p* < 0.001), increasing age, non-Dutch ethnicity and higher income had an independent positive impact on the uptake (Odds Ratio, OR: 2.71 (95% CI =2.41-3.05) *p* < 0.001; OR: 1.31 (95% CI = 1.04-1.66) *p* = 0.024; OR: 1.38 (95% CI = 1.16-1.65) *p* < 0.001 respectively), while being Protestant, multiparous and being from the eastern region compared with the western region had an independent negative impact on the uptake (OR: 0.25 (95% CI = 0.18-0.34) *p* < 0.001; OR: 0.63 (95% CI = 0.54-0.76) *p* < 0.001; OR: 0.31 (95% CI = 0.19-0.52) *p* < 0.001 respectively). Education, being Muslim and being from the northern region were not independently associated with the CT uptake.

**Table 4:** **Results of multilevel analysis; factors explaining variation in CT and FAS uptake among pregnant women participating in the DELIVER study**

	Model CT (n = 4,240)		Model FAS (n = 4,247)	
Predictor	OR (95% CI)	***p***-value	OR (95% CI)	***p***-value
**Higher education**			1.63 (1.07-1.49)	0.005
**Non-Dutch background**	1.31 (1.04-1.66)	0.024		
**Increasing age**	2.71 (2.41-3.05)	<0.001		
**Protestantism**	0.25 (0.18-0.34)	<0.001	0.37 (0.24-0.56)	<0.001
**Islam**			0.31 (0.20-0.48)	<0.001
**Above average income**	1.38 (1.16-1.65)	<0.001	1.66 (1.31-2.10)	<0.001
**East region**	0.31 (0.19-0.52)	<0.001		
**Multiparous**	0.63 (0.54-0.76)	<0.001	0.75 (0.62-0.89)	0.002

OR = odds ratio; random intercept for midwifery practices for both the CT model and the FAS model, additionally a random slope for Protestantism in the FAS model.

In the FAS model (*χ*^*2*^ (8, *N* = 4,247) = 113.95, *p* < 0.001), higher education and higher income had an independent positive impact on the uptake (OR: 1.63 (95% CI = .1.07-1.49) *p* =0.005; OR: 1.66 (95% CI = 1.31-2.10) *p* < 0.001 respectively), while being Protestant, Muslim and multiparous had an independent negative impact on the uptake (OR: 0.37 (95% CI = 0.24-0.56) *p* < 0.001; OR: 0.31 (95% CI = 0.20-0.48) *p* < 0.001; OR: 0.75 (95% CI = 0.62-0.89) *p* = 0.002 respectively). None of the regions independently influenced the FAS uptake. The random slope for Protestantism in the FAS model means that the uptake among Protestant women is different across the different practices. The three practices with the highest proportion of Protestant women (83%, 78% and 57% respectively) had the lowest CT and FAS uptakes; conversely, practices with the lowest percentages of Protestant women (3%, 4% and 5% respectively) had higher than average CT and FAS uptakes.

Finally, we performed subgroup analyses for the women with a western and women with a non-western non-Dutch ethnic background in the study population (respectively N = 401 and N = 400). The background characteristics of the women with a non-Dutch background that are related to the tests uptakes and information from the univariate logistic regression analyses are presented in Table 5. In comparison with the western women with a non-Dutch ethnic background, the non-western women were more religious, mostly Muslim, younger, had less education, were more likely to be from the western region of the Netherlands, multiparous, from the first generation, and have lower incomes. This subgroup had a higher percentage of limited proficiency in Dutch. The mean actual uptake among western non-Dutch women for the CT was 35% (141/401), and 94% for the FAS (377/401). The mean actual uptake among non-western non-Dutch women for the CT was 24% (94/400), and 84% for the FAS (337/400). Additionally, limited proficiency in Dutch among western non-Dutch women (27% (50/182)) was only found in the first generation. Limited proficiency in Dutch among non-western non-Dutch women was mostly found in the first generation (98% (118/121)).

**Table 5:** **Background characteristics and univariate logistic regression subgroup analyses of the uptake of the combined test (CT) and of the fetal anomaly scan (FAS) for the women with a western non-Dutch and with a non-western non-Dutch ethnic background participating in de DELIVER study**

	Western			Non-Western		
Determinant	Sample size n (%) ^a^	CT uptake	FAS uptake	Sample size n (%) ^a^	CT uptake	FAS uptake
		n (%) ^b^│ OR (95% CI)	n (%) ^b^│ OR (95% CI)		n (%) ^b^│ OR (95% CI)	n (%) ^b^│ OR (95% CI)
**Total**	401 (100%)	141 (35)	377 (94)	400 (100%)	94 (24)	337 (84)
**Religion**						
**None**	250 (63)	95 (38) │ 1	243 (97) │ 1	103 (26)	21 (20) │ 1	99 (96) │ 1
**Islam**	14 (3)	3 (21) │ 0.45 (0.12-1.64)	14 (100) │^f^	214 (54)	46 (22) │ 1.07 (0.60-1.91)	166 (78) │ 0.14 (0.05-0.40)^d^
**Protestantism**	54 (14)	9 (17) │ 0.33 (0.15-0.70)^c^	44 (82) │0.13 (0.05-0.35)^d^	27 (7)	5 (19) │ 0.89 (0.30-2.62)	23 (85) │ 0.23 (0.05-0.99)^c^
**Catholicism**	62 (16)	23 (37) │ 0.96 (0.54-1.71)	56 (90) │0.27 (0.09-0.83)^c^	23 (6)	8 (35) │ 2.08 (0.78-5.57)	21 (91) │ 0.42 (0.07-2.47)
**Other** ^**e**^	5 (1)	3 (60) │ 2.45 (0.40-14.92)	4 (80) │0.12 (0.01-1.19)	21 (5)	10 (48) │ 3.56 (1.33-9.91)^c^	20 (95) │ 0.81 (0.09-7.62)
**Would not say**	14 (3)	7 (50) │ 1.63 (0.56-4.80)	14 (100) │^f^	7 (2)	2 (29) │ 1.56 (0.28-8.62)	4 (57) │ 0.05 (0.01-0.33)^d^
**Age (years)**						
**≤ 30**	162 (41)	31 (19) │ 1	151 (93) │ 1	220 (55)	38 (17) │ 1	192 (87) │ 1
**31-35**	152 (38)	64 (42) │ 3.07 (1.85-5.10)^d^	146 (96)│1.77 (0.64-4.92)	131 (33)	36 (28) │ 1.82 (1.09-3.05)^c^	107 (82) │ 0.65 (0.36-1.18)
**≥36**	86 (21)	46 (54) │ 4.86 (2.73-8.65)^d^	79 (92)│ 0.82 (0.31-2.20)	49 (12)	20 (41) │ 3.30 (1.70-6.45)^d^	38 (78) │ 0.50 (0.23-1.10)
**Level of education**						
**Low**	45 (11)	12 (27) │ 1	43 (96) │ 1	117 (30)	28 (24) │ 1	89 (76) │ 1
**Medium**	139 (35)	40 (29) │ 1.11 (0.52-2.37)	131 (94)│ 0.76 (0.16-3.73)	136 (34)	24 (18) │ 0.68 (0.37-1.26)	113 (83)│ 1.55 (0.83-2.87)
**High**	215 (54)	88 (41) │ 1.91 (0.94-3.89)	201 (94) │ 0.67 (0.15-3.05	144 (36)	41 (29)│ 1.27 (0.72-2.21)	132 (92) │ 3.46 (1.67-7.17)^d^
**Income**						
**None to average**	121 (36)	30 (25) │ 1	114 (94) │1	215 (66)	48 (22) │ 1	174 (81) │ 1
**Above average**	217 (64)	86 (40) │ 2.00 (1.22-3.26)^c^	203 (94)│ 0.89 (0.35-2.27)	113 (34)	31 (27) │ 1.32 (0.78-2.20)	106 (94)│ 3.57 (1.55-8.24)^c^
**Region**						
**West**	141 (35)	65 (46) │ 1	133 (94) │ 1	229 (57)	58 (25)│ 1	192 (84) │ 1
**East**	65 (16)	22 (21) │ 0.31 (0.18-0.59)^d^	96 (92) │ 0.72 (0.26-1.99)	52 (13)	12 (16) │ 0.57 (0.29-1.13)	59 (80) │ 0.76 (0.39-1.48)
**South**	104 (26)	19 (29) │ 0.48 (0.26-0.91)^c^	61 (94) │ 0.92 (0.27-3.16)	74 (19)	14 (27) │ 1.09 (0.55-2.15)	45 (87) │ 1.23 (0.52-2.96)
**North**	91 (23)	35 (39) │ 0.73 (0.43-1.25)	87 (96) │ 1.31 (0.38-4.48)	45 (11)	10 (22) │ 0.84 (0.39-1.81)	41 (91) │ 1.98 (0.68-5.85)
**Parity**						
**Nulliparous**	198 (49)	74 (37) │ 1	187 (94) │ 1	163 (41)	45 (28) │ 1	147 (90) │ 1
**Multiparous**	203 (51)	67 (33) │ 0.83 (0.55-1.24)	190 (94) │ 0.86 (0.38-1.97)	237 (59)	49 (21) │ 0.68 (0.43-1.09)	190 (80) │ 0.44 (0.24-0.81)^c^
**Consanguinity**						
**No**	389 (99)	137 (35) │ 1	365 (94) │ 1	340 (90)	83 (24) │ 1	294 (87) │ 1
**Yes**	3 (1)	1 (33) │ 0.92 (0.83-10.23)	3 (100) │^f^	39 (10)	6 (15) │ 0.56 (0.23-1.39)	24 (62) │ 0.25 (0.12-0.51)^d^
**Generation**						
**First**	182 (45)	82 (45) │ 2.22 (1.47-3.38)^d^	169 (93) │0.69 (0.30-1.57)	250 (62)	74 (30) │ 2.73 (1.59-4.71)^d^	196 (78) │ 0.23 (0.11-0.49)^d^
**Second**	219 (55)	59 (27) │ 1	208 (95) │ 1	150 (36)	20 (13) │ 1	141 (94) │ 1
**Dutch language proficiency**						
**Excellent**	337 (87)	114 (34) │ 1	319 (95) │ 1	271 (69)	51 (19) │ 1	241 (89) │ 1
**Limited**	50 (13)	24 (48) │ 1.81 (1.00-3.29)^c^	46 (92) │ 0.65(0.21-2.00)	121 (31)	43 (36) │ 2.38 (1.47-3.85)^d^	90 (74) │ 0.36 (0.21-0.63)^d^

OR = Odds Ratio; CI = confidence interval; ^a^sample size varies due to missing data; valid percentages are shown; ^b^uptake percentage of population per category**;** ^c^p < 0.05; ^d^p < 0.001; ^e^Judaism, Buddhism, Hinduism; ^f^because of 100% score, no OR.

The results of the multivariate logistic regression subgroups analyses are presented in Table 6. In the CT model for western women of non-Dutch ethnic background (*χ*^*2*^ (7, *N* = 336) = 367.41, *p* < 0.001). Women who were Protestant, or living in the eastern region were significantly less likely to have the combined test (OR: 0.32 (95% CI =0.13-0.80) *p* =0.015; OR: 0.44 (95% CI = 0.21-0.93) *p* = 0.033 respectively). Older women, women with above average income, or women from the first generation were significantly more likely to have the CT (OR: 2.00 (95% CI = 1.44-2.78) *p* < 0.001; OR: 1.97 (95% CI = 1.12-3.45) *p* = 0.018; OR: 2.91 (95% CI = 1.75-4.85) *p* < 0.001 respectively). Being from the southern region and having limited proficiency in Dutch were not independently associated with the CT uptake.

**Table 6:** **Results of multivariate logistic regression subgroup analyses; factors explaining variation in the test uptake for women with western non-Dutch and with a non-western non-Dutch ethnic background participating in the DELIVER study**

	CT Model		FAS Model	
**Western**	(n = 336)		(n = 399)	
**Predictor**	OR (95% CI)	*p-*value	OR (95% CI)	*p-*value
Protestantism	0.32 (0.13-0.80)	0.015	0.13 (0.05-0.34)	<0.001
Catholicism			0.27 (0.09-0.81)	0.020
Increasing age	2.00 (1.44-2.78)	<0.001		
Above average income	1.97 (1.12-3.45)	0.018		
East Region	0.44 (0.21-0.93)	0.033		
First generation	2.91 (1.75-4.85)	<0.001		
**Non-Western**	(n = 392)		(n = 395)	
**Predictor**	OR (95% CI)	*p-*value	OR (95% CI)	*p-*value
Islam			0.37 (0.19-0.72)	0.003
Increasing age	1.73 (1.25-2.39)	0.001		
Higher education			1.47 (1.02-2.14)	0.041
Limited proficiency in Dutch	2.18 (1.34-3.56)	0.002		
First generation			0.27 (0.13-0.59)	0.001

OR = odds ratio; among women with a Western non-Dutch ethnic background: CT model: −2 log likelihood = 367.412, 69% correctly predicted; FAS model: −2 log likelihood = 164.002, 94% correctly predicted; among women with a non-Western non-Dutch ethnic background: CT model: −2 log likelihood = 408.956, 75% correctly predicted; FAS model: −2 log likelihood = 305.823, 85% correctly predicted.

Reference categories per variable: Protestantism: not being a Protestant; Catholicism: not being a Catholic; Islam: not being a Muslim; Increasing age: 30 years or younger; Above average income: below average or average income; Higher education: low education; East region: West Region; Limited proficiency in Dutch: excellent proficiency in Dutch; First generation: second generation.

Regarding the FAS uptake (*χ*^*2*^ (2, *N* = 399) = 164.00, *p* < 0.001), among western non-Dutch women, being Protestant, or Catholic had an independent impact on the uptake; women with these characteristics were significantly less likely to have the FAS (OR: 0.13 (95% CI = 0.05-0.34) *p* < 0.001; OR: 0.27 (95% CI = 0.09-0.81) *p* = 0.020 respectively).

In the CT model for non-western women of non-Dutch ethnic background (*χ*^*2*^ (3, *N* = 392) = 408.96, *p* < 0.001), older women or women with a limited proficiency in Dutch were significantly more likely to have the CT (OR: 1.73 (95% CI = 1.25-2.39) *p* < 0.001; OR: 2.18 (95% CI = 1.34-3.56) *p* = 0.002 respectively). Being from the first generation was not independently associated with the CT uptake.

Regarding the FAS uptake (*χ*^*2*^ (3, *N* = 395) = 305.82, *p* < 0.001), among non-western non-Dutch women, higher education had an independent positive impact on the uptake (OR: 1.47 (95% CI = .1.02-2.14) *p* =0.041), while being Muslim or from the first generation had an independent negative impact on the uptake (OR: 0.37 (95% CI = 0.19-0.72) *p* = 0.003; OR: 0.27 (95% CI = 0.13-0.59) *p* < 0.001 respectively). Being Protestant, having an income above average, and having a limited proficiency in Dutch were not independently associated with the FAS uptake.

## Discussion

This nationwide study focused on determining which factors are associated with the uptake of the prenatal congenital anomaly tests. Women were less likely to have the CT if they were multiparous, living in the eastern region, were native Dutch and identified themselves as Protestant; women were more likely to have the CT if they were older and had above average income. Women were less likely to have the FAS if they identified themselves as Protestant or Muslim and were multiparous, and women were more likely to have the FAS if they were more highly educated and had higher incomes. Contrary to our hypotheses, neither age nor ethnicity were associated with FAS uptake. Women in the subgroup of western non-Dutch ethnic background were less likely to have the CT if they identified themselves as Protestant and from the eastern region; women were more likely to have the CT if they were older, had higher incomes and from the first generation. Western women with a non-Dutch background were less likely to have the FAS if they identified themselves as Protestant or Catholic. Among the subgroup of non-western women with a non-Dutch ethnic background, these women were more likely to have the CT if they were older and had limited proficiency in Dutch. Non-western women with a non-Dutch background were less likely to have the FAS if they identified themselves as Muslim and from the first generation; women were more likely to have the FAS if they were higher educated.

The mean CT uptake in our study (23%) was a little bit lower than reported in previous studies (27%) [6, 7]. An explanation for the lower uptake in our study could be, that some participants who indicated that they did not have the CT may have entered prenatal care after the first trimester and thus been ineligible for the test; on the other hand, pregnant women in the Netherlands enter midwifery care around 9 weeks of gestation [29]. The mean CT uptake in our study is considerably lower than in other EU countries, such as the UK (60%) and Denmark (90%), where the CT is routinely offered as part of prenatal care [1–3]. The routine offering may act to normalise this screening test and increase its acceptance among pregnant women and partners. Previous studies have reported various reasons given by Dutch women for not opting for the CT: the test characteristics, high costs and attitudes towards Down syndrome [8, 10]. Religious women, for example, are more likely to accept a child with Down syndrome and less likely to terminate their pregnancy [30]. As in previous studies, we found a strong association between religious background and declining the CT [10–18]. An additional finding in our study is that the influence of religious background is faith-specific. Both Protestant and Muslim women show a much lower test uptake compared to Catholic women, who are similar to the non-religious group. In the Dutch context, this may be explained by Dutch Catholics generally having a more liberal attitude towards religious doctrines compared to Dutch Protestants [31]. The three midwifery practices with the lowest CT uptake were located in a predominantly orthodox Protestant region, known as the Dutch Bible Belt (Table 2), partly located in the eastern region, where for example the vaccination rate of children for infectious diseases is also low [31, 32].

Our study confirmed the variation in CT uptake across the regions that have previously been reported [6–8]. We found clustering of data in the midwifery practices, demonstrating that in addition to regional variance there were large differences in CT uptake (4% to 48%) amongst practices even within regions. Part of this variance may be accounted for by the different ways in which health professionals present prenatal congenital anomaly screening tests to pregnant women [33]; part of this variance may be due to the aforementioned religious variations found across regions. Further investigation is needed into the underlying causes of differences at the practice level.

Our study confirms earlier findings that women who were older than 30 were significantly more likely to have a CT [8, 10, 15] and is likely to reflect increased concerns about Down syndrome among older pregnant women. As is well known, the probability of bearing a child with Down Syndrome increases with the age of the mother [27]. Perhaps fewer younger women choose the CT because women younger than 36 must pay for the CT. This co-payment requirement may explain our study findings that women with a higher income have a significantly higher uptake [8].

Another explanation for the relatively low CT uptake in the Netherlands could be that women who decline the CT do routinely receive an ultrasound at the same time to confirm the pregnancy and to calculate the due date. Unlike the case of the CT, women who decline the FAS do not receive an alternative routine second-trimester ultrasound. This could partly explain the differences between the low CT uptake and the high FAS uptake, as we do know that women like to see their baby on an ultrasound scan and that they experience a scan as an encounter with the baby [34].

The mean FAS (90%) uptake in our study was comparable to the findings from earlier studies in the Netherlands [6, 7]. The mean FAS uptake in our study is lower compared to EU countries such as Sweden (99-100%) [10]. If we compare the Netherlands to other countries, an explanation of the high uptake in Sweden could be that the FAS was introduced by adding it to a routine second trimester ultrasound for fetal and placental measurements. Since the introduction of the screening programme in the Netherlands, women are offered the second-trimester ultrasound for detecting structural anomalies and fetal and placental measurements happen to be recorded at the same time; however, it was introduced primarily as screening for neural tube defects [35]. Women declining the FAS do not receive a routine ultrasound in the second trimester, which means that additional information that might be important in managing the pregnancy is not obtained. Several studies have stressed the importance of fetal growth measurements and placenta evaluation during the second trimester; fetuses smaller than expected between 18 and 22 weeks gestation are at risk of preterm birth and perinatal death, and abnormalities of the placenta can predict serious complications such as intrauterine growth restriction, preterm birth and preeclampsia [36–39]. The introduction of the FAS seems to be related to a decrease in perinatal mortality [40, 41]. Recently, Schoonen *et al.* developed an instrument to determine client’s decision-relevant knowledge about the FAS that only addresses determination of fetal anomalies and does not address fetal and placental measurements [32]. In view of these considerations, we suggest that the second-trimester ultrasound for fetal and placental measurements should be offered routinely, and additionally to detect anomalies following an opt-in system at the same time.

Notwithstanding a lower rate of access to healthcare amongst immigrant women [21, 22], surprisingly, in our study non-western women with a non-Dutch background were more likely to have a CT compared to women from Dutch origin (respectively 24% and 22%). Contrary to our findings, Fransen *et al.* found a lower CT uptake among non-western women (around 12%) [13]. The differences in results could probably be find in a different study design. Fransen *et* al. used data of one specific Medical Diagnostic Centre in a high density immigrant area, and in our national study, we collected data by purposive sampling; furthermore, the percentage non-Dutch women in our sample was lower compared to the non-Dutch pregnant population and the other study (respectively 17% and 34%) [13]. Also surprisingly, in our study non-western women with a non-Dutch background with limited proficiency in Dutch were more likely to have a CT. An explanation of the higher uptake could be that the women did not have an adequate understanding of the CT [42]. A practical implication of inadequate understanding of the tests indicates that caregivers should make sure that all clients fully understand the information about the screening tests. In addition to other plausible reasons, ninety-eight percent of the non-western women with a limited proficiency in Dutch were from the first generation and it could be that raising an disabled child may be viewed as complicating already complex lives and may be expensive. On the other hand, non-western women with limited proficiency had a significantly lower FAS uptake compared to non-western women with excellent proficiency (74% and 89% respectively). An explanation why more non-western women with limited Dutch proficiency choose early screening but fewer choose second-trimester screening could be found in the religious background. More than 50% of the non-western in our study were Muslim and from a religious perspective, Muslim women may prefer earlier screening because termination of pregnancy in the case of confirmed serious anomalies is permissible up to the 120^th^ day after conception, that is at 19 weeks’ gestation, but not later in pregnancy [18, 43, 44].

Finally, a remark on recent developments in prenatal anomaly screening. Since April 2014, the non-invasive prenatal test (NIPT) is implemented in the Dutch prenatal anomaly screening in a nationwide study context [45]. The NIPT is offered after a positive result of the CT and before a diagnostic test such as an amniocentesis. Only women who are at high risk, e.g. have a child with Down’s syndrome in a previous pregnancy, can choose to have the NIPT without a CT; women who are 36 years or older must first have a CT. Therefore, understanding the determinants of the CT uptake takes on new importance. With the implementation of the NIPT, prenatal anomaly screening has now four steps: 1) CT, 2) follow up positive CT results with the NIPT, 3) follow up positive NIPT result with diagnostic tests which carry an associated 0.5% risk of miscarriage, and 4) when a positive finding is confirmed, to terminate the pregnancy before 24 weeks’ gestation or prepare for having a child with an anomaly.

As far as we know, this is the first nationwide Dutch study to examine which socio-demographic factors were associated with the uptake of prenatal congenital anomaly screening tests in a low-risk population and to provide insight into the similarities and differences between the uptake of the two screening tests. No data was collected on factors such as anomalies in previous pregnancies and family history of genetic diseases and this might bias the results; on the other hand, in general pregnant women at risk for congenital anomalies usually receive prenatal care from obstetricians from the start. Study participants were representative with respect to age and parity, but highly educated women and native Dutch women were overrepresented; the limitations of the study are discussed at length by Manniën *et al*. [24]. The high proportion of highly educated women may have resulted in over-reporting of the average CT and FAS uptake, while the overrepresentation of native Dutch women might have resulted in underreporting of the average CT uptake. The low response of some midwifery practices, the unequal distribution of the participants across practices and the possible differences in how the practices presented the tests may limit the generalizability of the findings [24]. Because of the small numbers of the secondary analyses among the subgroups, the results should be interpreted cautiously, and further research among western and non-western non-Dutch women with a larger sample size will be important to enhance the generalizability of the results. Additional research is also needed on the different ways in which the prenatal anomaly tests are presented.

## Conclusion

Our study found that different socio-demographic factors were associated with the uptake of the CT and the FAS. Multiparity and some faiths were negatively associated with both screening tests and higher income was positively associated with both tests. Non-Dutch ethnic background and increasing age were positively associated with the CT uptake while there was a negative association with living in the eastern region. The level of education was positively associated with the FAS uptake. Also, limited proficiency in Dutch was positively associated with the CT uptake among non-western women. Our findings help to explain some differences between women choosing or declining early and late screening, but not the large variation in the test uptake among practices, nor between the Netherlands and other countries.

## Abbreviations

CT: Combined test
FAS: Fetal anomaly scan.
